# Quantum chemical assessment of benzimidazole derivatives as corrosion inhibitors

**DOI:** 10.1186/1752-153X-8-21

**Published:** 2014-03-27

**Authors:** Hasan R Obayes, Ghadah H Alwan, Abdul Hameed MJ Alobaidy, Ahmed A Al-Amiery, Abdul Amir H Kadhum, Abu Bakar Mohamad

**Affiliations:** 1 Applied Chemistry Division, Applied Science Department, University of Technology, Baghdad, Iraq; 2 Ministry of Sciences and Technology, Industrial Research & Development Directorate, Industrial Applications Center, Baghdad, Iraq; 3 Environmental Research Center, University of Technology (UOT), Baghdad 10001, Iraq; 4 Department of Chemical & Process Engineering, Universiti Kebangsaan Malaysia (UKM), Bangi, Selangor 43000, Malaysia

**Keywords:** Benzimidazole, B3LYP, Corrosion, DFT, Inhibitor

## Abstract

**Background:**

The majority of well-known inhibitors are organic compounds containing multiple bonds and heteroatoms, such as O, N or S, which allow adsorption onto the metal surface. These compounds can adsorb onto the metal surface and block active surface sites, reducing the rate of corrosion.

**Results:**

A comparative theoretical study of three benzimidazole isomers, benzimidazole (BI), 2-methylbenzimidazole (2-CH_3_-BI), and 2-mercaptobenzimidazole (2-SH-BI), as corrosion inhibitors was performed using density functional theory (DFT) with the B3LYP functional basis set.

**Conclusions:**

Nitro and amino groups were selected for investigation as substituents of the three corrosion inhibitors. Nitration of the corrosion inhibitor molecules led to a decrease in inhibition efficiency, while reduction of the nitro group led to an increase in inhibition efficiency. These aminobenzimidazole isomers represent a significant improvement in the inhibition efficiency of corrosion inhibitor molecules.

## Introduction

Corrosion is an electrochemical process by which metallic structures are destroyed gradually through anodic dissolution [1]. Protection of metallic surfaces can be achieved by the addition of specific compounds known as corrosion inhibitors [2]. Among the numerous corrosion prevention measures available, corrosion inhibitors, which have the advantages of economy, high-efficiency, and facile and feasible use, have been widely applied in various fields. As the importance of environmental protection has become increasingly recognized, the development of new green corrosion inhibitors has received increasing attention [3-5]. A variety of organic compounds containing heteroatoms (N, O, S) that can donate electron pairs have been used to inhibit brass corrosion in various aggressive electrolytes [6-11]. The use of organic inhibitors for preventing corrosion is a promising alternative. These inhibitors are usually adsorbed on the metal surface by the formation of a coordinate covalent bond (chemical adsorption) or an electrostatic interaction between the metal and inhibitor (physical adsorption) [12]. This adsorption produces a uniform film on the metal surface, which reduces or prevents contact with the corrosive medium [13]. Because organic inhibitors act by adsorption on the metal surface, the efficiency of these compounds depends strongly on their ability to form complexes with the metal [14]. Both *p* electrons and polar groups containing sulfur, oxygen and nitrogen are fundamental characteristics of this type of inhibitor. The polar functional groups serve as the chelation center for chemical adsorption [15]. Considerable effort has been devoted to studying the metallic corrosion inhibition properties of benzimidazole and its derivatives [16-20]. Benzimidazole is a heterocyclic aromatic organic compound with a bicyclic structure comprising fused benzene and imidazole rings [21]. The hydrogen atoms on the rings can be substituted by other groups or atoms. Some derivatives of benzimidazole are excellent corrosion inhibitors for metals and alloys in acidic solution; the level of inhibition varies with substituent groups and substituent positions on the imidazole ring [22-26]. The effects of the molecular structure on chemical reactivity have been studied extensively [27-31]. Density functional theory (DFT) was recently successfully applied to describe the structural importance of corrosion inhibitors and their adsorption efficiency on metal surfaces [32,33]. As part of the development of novel, more efficient organic corrosion inhibitors, several quantum-chemistry studies have been performed that relate inhibition efficiency to the molecular properties of the different types of compounds. The molecular structure and the electronic parameters, which can be obtained from theoretical calculations and include the HOMO (highest occupied molecular orbital) energy, the LUMO (lowest unoccupied molecular orbital) energy, and the energy of the gap, influence the inhibitor activity as well as reactivity, which can be treated by HSAB theory [34-42]. The aim of this work is to elucidate the electron configuration of benzimidazole (BI), 2-methylbenzimidazole (2-CH3-BI) and 2-mercaptobenzimidazole (2-SH-BI) inhibitors using DFT and determine the relationship between molecular structure and inhibition efficiency. The established correlation will facilitate the design and synthesis of new inhibitors with improved inhibition efficiency.

### The calculation method

To calculate the ground-state geometries, Gaussian 03, Revision C.01 [43] was optimized to a local minimum without symmetry restrictions using the valence and polarization basis set (6-31G++(d,p)) [44,45]. A combination of the Becke three-parameter hybrid (B3) [46,47] exchange functional and the Lee-Yang-Parr (LYP) [48] correlation functional (B3LYP) [49,50], a version of the (DFT) method [51,52] was used to determine all optimized geometries, HOMO energies (EHOMO), LUMO energies (ELUMO), and physical properties for the molecules in this study.

## Results and discussion

Two different groups were chosen as substituents of the corrosion inhibitor molecules BI, 2-CH3-BI, and 2-SH-BI to include the most important electronic effects. The first group (nitro (-NO2)) is a strong acceptor, while the second (amino (-NH2)) is a strong donor. The nitration of corrosion inhibitor molecules yielded four models for each of the corrosion inhibitor molecules, and the same number of models was obtained for the reduced nitro group [53].

### Benzimidazole (BI)

**Figure 1:**
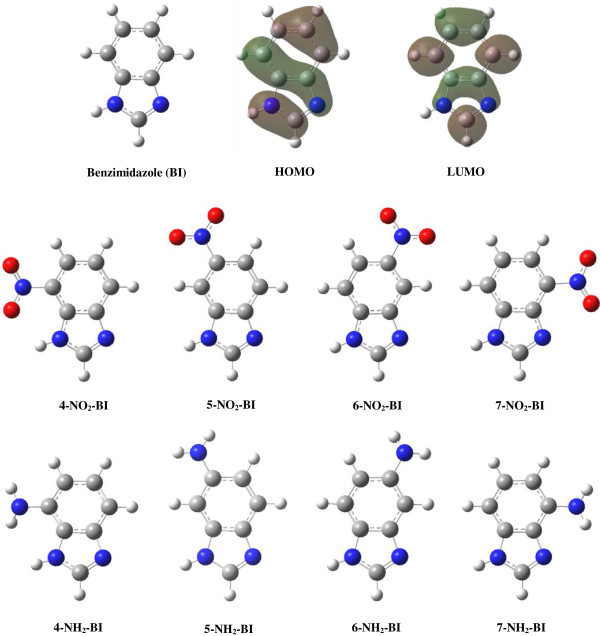
B3LYP/6-31G++(d,p) optimized geometries, HOMO and LUMO of benzimidazole (BI) and the optimized geometries of the eight models.

The four positions of the nitro group substituent on the benzene ring in BI were C-4, C-5, C-6 and C-7. These positions make the same contribution to both the HUMO and LUMO levels with a small difference, as shown in Figure 1. Figure 1 also shows the structures of the optimized geometries for BI and the models studied. Table 1 presents the EHOMO, ELUMO and energy gap values for (BI) and all models. The ionization potential (I) and the electron affinity (A) were calculated by application of Koopman’s theorem [54]. This theorem establishes a relationship between the energies of the HOMO and the LUMO and the ionization potential and electron affinity, respectively.

**Table 1:** Quantum-chemical parameters for benzimidazole (BI) and eight models as determined by DFT at the B3LYP/6-31G++ (d,p) level

**Molecules**	**Total Energy a.u.**	**EHOMO eV**	**ELUMO eV**	**Gap energy (ELUMO - EHOMO) eV**	**Ionization potential (I)**	**Electron affinity (A)**
BI	-379.9673	-6.4567	-0.8778	5.5789	6.4567	0.8778
4-NO2-BI	-584.5358	-7.3076	-3.1016	4.2060	7.3076	3.1016
4-NH2-BI	-435.3409	-5.7838	-0.7295	5.0543	5.7838	0.7295
5-NO2-BI	-584.5321	-7.2878	-2.8316	4.4562	7.2878	2.8316
5-NH2-BI	-435.3418	-5.5060	-0.6514	4.8546	5.5060	0.6514
6-NO2-BI	-584.5319	-7.2546	-2.6667	4.5879	7.2546	2.6667
6-NH2-BI	-435.3409	-5.4986	-0.7600	4.7386	5.4986	0.7600
7-NO2-BI	-584.5220	-7.2404	-2.7045	4.5359	7.2404	2.7045
7-NH2-BI	-435.3472	-5.4235	-0.4863	4.9372	5.4235	0.4863

I = - EHOMO

A = - ELUMO

Table 2 presents the calculated values of inhibition efficiency % for BI and eight models, which were determined using the following formula:

$$I_{\mathrm{add}.}\%=\frac{I_{\mathrm{BI}}-I_{x-\mathrm{BI}}}{I_{\mathrm{BI}}}\times 100\%$$

$$Ie_{\mathrm{add}.}\%=I_{\mathrm{add}.}\%\;\times Ie_{\mathrm{BI}}\%$$

$$Ie_{\mathrm{theor}.}\%=Ie_{\mathrm{BI}}\%+Ie_{\mathrm{add}.}\%\;$$

**Table 2:** The calculated inhibition efficiency % of benzimidazole (BI) and eight models

**Molecules**	***I***_***add*** **.**_**%**	***Ie***_***add*** **.**_**%**	**Inhibition efficiency %**	
			**Theoretical (*Ie***_***theor*** **.**_**)**	**Experimental**
BI	0	0	73.800	73.8
4-NO2-BI	-13.178	-9.725	64.075	----
4-NH2-BI	+10.422	+7.691	81.491	----
5-NO2-BI	-12.872	-9.500	64.300	----
5-NH2-BI	+14.724	+10.866	84.666	----
6-NO2-BI	-12.358	-9.120	64.680	----
6-NH2-BI	+14.839	+10.951	84.751	----
7-NO2-BI	-12.138	-8.958	64.842	----
7-NH2-BI	+16.002	+11.809	85.609	----

Where *I*_*add*._% is the percentage ionization potential of the additive for model (*x* - *BI*), *Ie*_*add*._% is the inhibition efficiency %of the additive, and *Ie*_*theor*._% is the theoretically calculated percentage inhibition efficiency.

These results demonstrate that the nitration of corrosion inhibitor molecules lead to a decrease in inhibition efficiency; the most efficient inhibitor was model (4-NO2-BI), which displayed an inhibition efficiency of 64.075%. By contrast, reduction of the nitro group led to an increase in inhibition efficiency; the most efficient inhibitor was model (7-NH2-BI), which displayed an inhibition efficiency of 85.609%. The inhibition efficiency of BI was 73.8%. These results represent a significant improvement in the inhibition efficiency of BI.

### Methylbenzimidazole (2-CH3-BI)

**Figure 2:**
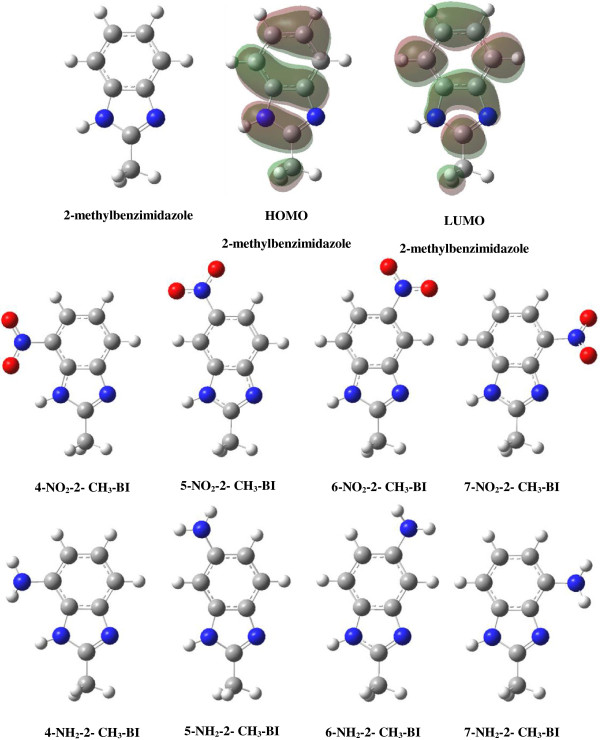
**B3LYP/6-31G++(d,p) optimized geometries, HOMO and LUMO of 2-methylbenzimidazole (2-CH**_**3**_**-BI) and the optimized geometries of eight models.**

The nitro group can be substituted at positions C-4, C-5, C-6 and C-7 on the benzene ring in 2- CH3-BI. These positions make the same contributions to the HUMO and LUMO levels, with the exception of position C-7, which is poor in the HUMO level as shown in Figure 2. Figure 2 also presents the structures of the optimized geometries for 2-CH3-BI and the studied models. Table 3 presents the EHOMO, ELUMO and energy gap values for (2-CH3-BI) and all models. Koopmans’ theorem was used to calculate I and A [50].

**Table 3:** **Quantum-chemical parameters for 2-methylbenzimidazole (2-CH**_**3**_**-BI) and eight models obtained using DFT at the B3LYP/6-31G++ (d,p) level**

**Molecules**	**Total Energy a.u.**	**EHOMO eV**	**ELUMO eV**	**Gap energy (ELUMO - EHOMO ) eV**	**Ionization potential (I)**	**Electron affinity (A)**
2-CH3-BI	-419.3013	-6.2611	-0.6963	5.5648	6.2611	0.6963
4-NO2-2-CH3-BI	-623.8708	-7.0401	-2.9818	4.0583	7.0401	2.9818
4-NH2-2-CH3-BI	-474.6748	-5.6826	-0.6966	4.9860	5.6826	0.6966
5-NO2-2-CH3-BI	-623.8672	-7.1062	-2.7092	4.3970	7.1062	2.7092
5-NH2-2-CH3-BI	-474.6754	-5.3555	-0.5034	4.8521	5.3555	0.5034
6-NO2-2-CH3-BI	-623.8669	-7.0418	-2.5557	4.4861	7.0418	2.5557
6-NH2-2-CH3-BI	-474.6746	-5.3721	-0.5992	4.7729	5.3721	0.5992
7-NO2-2-CH3-BI	-623.8576	-6.9794	-2.4945	4.4849	6.9794	2.4945
7-NH2-2-CH3-BI	-474.6807	-5.3220	-0.4743	4.8477	5.3220	0.4743

Table 4 presents the calculated values of inhibition efficiency % for 2-CH3-BI and eight models, which were determined using the following formula:

$$I_{\mathrm{add}.}\%=\frac{I_{2-CH_{3}-\mathrm{BI}}-I_{x-2-CH_{3}-\mathrm{BI}}}{I_{2-CH_{3}-\mathrm{BI}}}\times 100\%$$

$$Ie_{\mathrm{add}.}\%=I_{\mathrm{add}.}\%\times Ie_{2-CH_{3}-\mathrm{BI}}\%$$

$$Ie_{\mathrm{theor}.}\%=Ie_{2-CH_{3}-\mathrm{BI}}\%+Ie_{\mathrm{add}.}\%\;$$

**Table 4:** **Calculated inhibition efficiency % for 2-methylbenzimidazole (2-CH**_**3**_**-BI) and eight models**

**Molecules**	***I***_***add*** **.**_**%**	***Ie***_***add*** **.**_**%**	**Inhibition efficiency %**	
			**Theoretical (*Ie***_***theor*** **.**_**%)**	**Experimental**
2-CH3-BI	0	0	76.300	76.3
4-NO2-2-CH3-BI	-12.442	-9.493	66.807	----
4-NH2-2-CH3-BI	+9.240	+7.050	83.350	----
5-NO2-2-CH3-BI	-13.498	-10.299	66.001	----
5-NH2-2-CH3-BI	+14.464	+11.036	87.336	----
6-NO2-2-CH3-BI	-12.469	-9.514	66.786	----
6-NH2-2-CH3-BI	+14.199	+10.834	87.134	----
7-NO2-2-CH3-BI	-11.472	-8.753	67.547	----
7-NH2-2-CH3-BI	+14.999	+11.444	87.744	----

Where *I*_*add*._% is the percentage of ionization potential additive for model (*x* - 2 - *CH*_3_ - *BI*), *Ie*_*add*._% is the percentage of inhibition efficiency additive, and *Ie*_*theor*._% is the theoretical calculated percentage of inhibition efficiency.

These results demonstrate that the nitration of corrosion inhibitor molecules decreases the inhibition efficiency; the highest inhibition efficiency, 66.001%, was obtained for the model (5-NO2-2-CH3-BI). By contrast, reduction of the nitro group led to an increase in inhibition efficiency; the highest inhibition efficiency, 87.44%, was observed for the model (7-NH2-2-CH3-BI). The inhibition efficiency of 2-CH3-BI was 76.3%. These results represent a significant improvement in the inhibition efficiency of 2-CH3-BI.

### Mercaptobenzimidazole (2-SH-BI)

**Figure 3:**
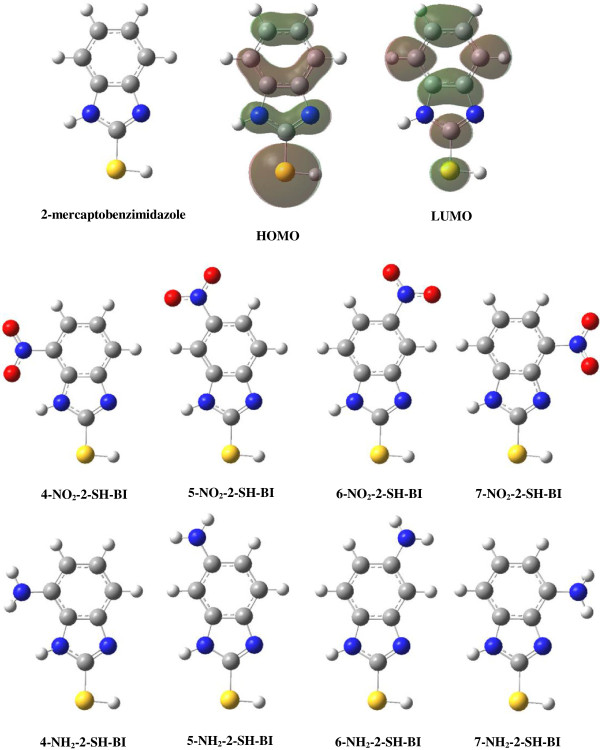
B3LYP/6-31G++(d,p) optimized geometries, HOMO and LUMO of 2-mercaptobenzimidazole (2-SH-BI) and the optimized geometries of eight models.

The positions on the benzene ring in 2-SH-BI that were substituted with nitro groups were C-4, C-5, C-6 and C-7. These positions make the same contribution to both the HUMO and LUMO levels, with a small difference as shown in Figure 3. Figure 3 also shows the structures of the optimized geometries for 2-SH-BI and the studied models. Table 5 presents the EHOMO, ELUMO and energy gap values for 2-SH-BI and all models. Koopman’s theorem was used to calculate I and A [50].

**Table 5:** Quantum-chemical parameters for 2-mercaptobenzimidazole (2-SH-BI) and eight models determined using DFT at the B3LYP/6-31G++ (d,p) level

**Molecules**	**Total Energy a.u.**	**EHOMO eV**	**ELUMO eV**	**Gap energy eV**	**Ionization potential (I)**	**Electron affinity (A)**
2-SH-BI	-778.1839	-6.1585	-0.8242	5.3343	6.1585	0.8242
4-NO2-2-SH-BI	-982.7523	-6.7906	-3.0743	3.7163	6.7906	3.0743
4-NH2-2-SH-BI	-833.5576	-5.8358	-0.7747	5.0611	5.8358	0.7747
5-NO2-2-SH-BI	-982.7492	-6.9024	-2.7856	4.1168	6.9024	2.7856
5-NH2-2-SH-BI	-833.5580	-5.3873	-0.6332	4.7541	5.3873	0.6332
6-NO2-2-SH-BI	-982.7486	-6.8461	-2.6580	4.1881	6.8461	2.6580
6-NH2-2-SH-BI	-833.5575	-5.4698	-0.7189	4.7509	5.4698	0.7189
7-NO2-2-SH-BI	-982.7398	-6.7707	-2.6172	4.1535	6.7707	2.6172
7-NH2-2-SH-BI	-833.5630	-5.4480	-0.6713	4.7767	5.4480	0.6713

Table 6 presents the calculated values of inhibition efficiency % for 2-SH-BI and eight models, which were determined using the following formula:

$$I_{\mathrm{add}.}\%=\frac{I_{2-\mathrm{SH}-\mathrm{BI}}-I_{x-2-\mathrm{SH}-\mathrm{BI}}}{I_{2-\mathrm{SH}-\mathrm{BI}}}\times 100\%$$

$$Ie_{\mathrm{add}.}\%=I_{\mathrm{add}.}\%\times Ie_{2-\mathrm{SH}-\mathrm{BI}}\%$$

$$Ie_{\mathrm{theor}.}\%=Ie_{2-\mathrm{SH}-\mathrm{BI}}\%+Ie_{\mathrm{add}.}\%\;$$

**Table 6:** Calculated inhibition efficiency % for 2-mercaptobenzimidazole (2-SH-BI) and eight models

**Molecules**	***I***_***add*** **.**_**%**	***Ie***_***add*** **.**_**%**	**Inhibition efficiency %**	
			**Theoretical (*Ie***_***theor*** **.**_**%)**	**Experimental**
2-SH-BI	0	0	90.1	90.1
4-NO2-2-SH-BI	-10.264	-9.248	80.852	----
4-NH2-2-SH-BI	+5.240	+4.721	94.821	----
5-NO2-2-SH-BI	-12.079	-10.883	79.217	----
5-NH2-2-SH-BI	+12.522	+11.282	101.382	----
6-NO2-2-SH-BI	-11.165	-10.060	80.040	----
6-NH2-2-SH-BI	+11.183	+10.076	100.176	----
7-NO2-2-SH-BI	-9.9407	-8.950	81.150	----
7-NH2-2-SH-BI	+11.537	+10.394	100.494	----

Where *I*_*add*._% is the percentage of ionization potential additive for model (*x* - 2 - *SH* - *BI*), *Ie*_*add*._% is the percentage of inhibition efficiency additive, and *Ie*_*theor*._% is the theoretically calculated percentage of inhibition efficiency.

These results demonstrate that the nitration of corrosion inhibitor molecules led to a decrease in inhibition efficiency; the highest inhibition efficiency, 79.217%, was obtained for the model (5-NO2-2-SH-BI). By contrast, reduction of the nitro group led to an increase in inhibition efficiency; the highest inhibition efficiency, 101.382%, was obtained for the model (5-NH2-2-SH-BI). The inhibition efficiency of 2-SH-BI was 90.1%. These results represent a significant improvement in the inhibition efficiency of 2-SH-BI.

## Conclusions

DFT quantum-chemical calculations established a correlation between parameters related to electronic structure and the corrosion inhibition potential of the three corrosion inhibitor molecules BI, 2-CH3-BI, and 2-SH-BI, as well as eight models for each inhibitor molecule. Most of the molecular parameters calculated at the B3LYP/6-311G++(d,p) level indicated that the nitration of corrosion inhibitor molecules led to a decrease in inhibition efficiency, while reduction of the nitro group led to an increase in inhibition efficiency. These results represent a significant improvement in inhibition efficiency compared to previously reported corrosion inhibitor molecules. An excellent correlation between inhibition efficiency and the studied models was obtained, confirming the reliability of the method employed.

## Competing interests

The authors declare that there is no conflict of interests regarding the publication of this paper.

## Authors’ contribution

HO carried out DFT studies. GA carried out the screening studies on corrosion. AA carried out the calculation of inhibition efficiency. AA carried out the computational experiments. AK conceived of the study. AM draft the manuscript. All authors read and approved the final manuscript.
